# Acquired Amegakaryocytic Thrombocytopenia and Pure Red Cell Aplasia in Thymoma

**DOI:** 10.1155/2018/5034741

**Published:** 2018-03-11

**Authors:** Sumit Dahal, Eliza Sharma, Suyash Dahal, Binav Shrestha, Bikash Bhattarai

**Affiliations:** ^1^Interfaith Medical Center, Brooklyn, NY, USA; ^2^Maimonides Medical Center, Brooklyn, NY, USA; ^3^KIST Medical College and Teaching Hospital, Lalitpur, Nepal

## Abstract

Association of thymoma with myasthenia gravis, pure red cell aplasia, and aplastic anemia is well documented. However, thymoma complicated by acquired amegakaryocytic thrombocytopenia (AAMT) is rarely reported. Here, we present a case of a 60-year-old male with past medical history of recurrent invasive thymoma who presented with cough and blood in sputum. He was found to have severe normocytic normochromic anemia and thrombocytopenia that did not improve with intravenous steroids or multiple transfusions of red cells and platelets. Subsequent bone marrow biopsy showed severely depleted megakaryocytes and erythroid precursor cells with relative myeloid hyperplasia suggestive of amegakaryocytic thrombocytopenia and red cell aplasia. He was started on oral cyclosporine but subsequently developed leukopenia and refused any further treatment or diagnostic procedures and left the hospital against medical advice. AAMT, thus, may be a very early presentation of impending aplastic anemia, and treating physicians need to be aware of this entity.

## 1. Introduction

Thymomas are rare tumors with an annual incidence of 0.15 cases per 100,000 person-years in the United States [1]. They are, however, the most common tumor of the anterior mediastinum and are associated with a variety of paraneoplastic syndromes. Their association with myasthenia gravis, pure red cell aplasia (PRCA), and aplastic anemia is well documented [2–4]. Cases of thymoma complicated by acquired amegakaryocytic thrombocytopenia (AAMT) are, however, rarely reported. Here, we present a case of treated recurrent thymoma associated with development of concurrent PRCA and AAMT.

## 2. Case Report

**Figure 1:**
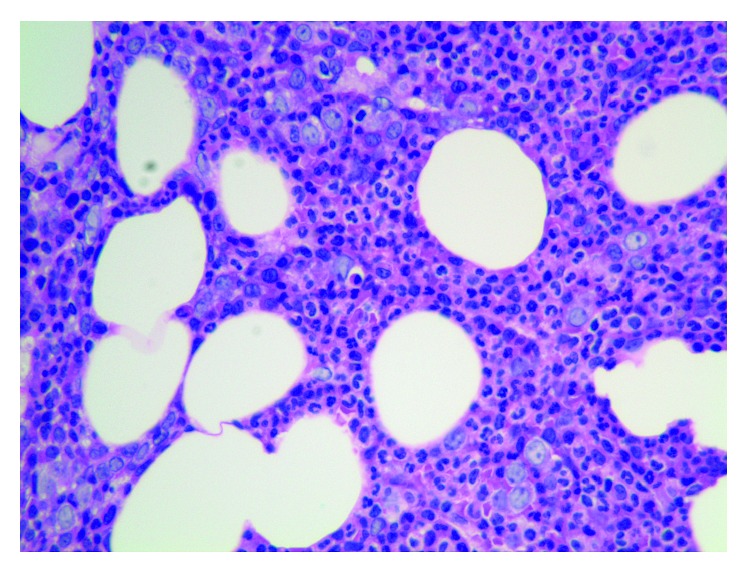
Bone marrow biopsy.

A 60-year-old male presented with cough and blood in sputum for three days. While he denied any fever, chills, chest pain, night sweats, and change in appetite or weight recently, review of system was significant for exertional shortness of breath and dizziness for the last few months. He also denied bleeding from any other site or bluish patches in the skin. His past medical history was significant for chronic hepatitis C, recurrent invasive thymoma, type 4 pulmonary hypertension due to chronic thromboembolism, and an episode of deep vein thrombosis. He was initially diagnosed with Masaoka stage II, World Health Organization type B1 thymoma, and underwent surgical resection for the same. There was, however, local recurrence of his disease three years later, and he was treated with chemotherapy and radiation. His only current home medications were sofosbuvir and velpatasvir for chronic hepatitis C. At presentation, his blood pressure was 104/69 mm of Hg, temperature was 98 F, pulse rate was 87 beats per minute, and oxygen saturation was 98% in room air. Physical examination was unremarkable except for marked conjunctival pallor. Initial lab work showed total white blood cell of 8,500/*μ*L (normal 4,500–11,000/*μ*L), total red blood cell of 1.88 million/*μ*L (normal 4–5.7 million/*μ*L), hemoglobin of 5.8 g/dL (normal 13–17 g/dL), mean corpuscular volume of 88.4 fL (normal 80–100 fL), mean corpuscular hemoglobin concentration of 34.6 g/dL (normal 30.5–36 g/dL), and a platelet count of 16,000/*μ*L (normal 130,000–400,000/*μ*L), serum potassium of 4.7 mmol/L (normal 3.6–5.1 mmol/L), blood urea nitrogen of 15 mg/dL (normal 8–20 mg/dL), serum creatinine of 0.8 mg/dL (normal 0.4–1.3 mg/dL), total serum bilirubin of 1.4 mg/dL (normal 0.3–1.2 mg/dL), aspartate aminotransferase of 25 IU/L (normal 15–41 IU/L), and alanine aminotransferase of 15 IU/L (normal 17–63 IU/L). Subsequent tests revealed a low reticulocyte of 0.09% (normal 0.5–2%) with a reticulocyte index of 0.02 and a lactate dehydrogenase of 168 IU/L (normal 98–192 IU/L). With the chest imaging showing stable postoperative changes in the upper left hemothorax with stable chronic pulmonary embolism, and the subsequent blood cultures, sputum cultures, and 3 sets of acid fast bacillus smears returning negative, the hemoptysis was attributed to severe thrombocytopenia, possibly due to hepatitis C-induced immune-complex mediated thrombocytopenia or myelodysplasia secondary to prior chemotherapy. The patient was transfused with 2 units of packed red cells, 1 unit of single donor platelets and started on intravenous methylprednisolone at a dose of 1,000 mg daily for three days followed by 125 mg daily. The patient had no further episode of hemoptysis or bleeding from any other site; however, his RBCs (2.46 million/*μ*L), Hb (7.2 g/dL), Hct (21.5%), and platelets (26,000/*μ*L) continued to be severely low even after two weeks of steroid therapy. A bone marrow biopsy was done which revealed hypercellular marrow with severely depleted megakaryocytes and erythroid precursor cells and relative myeloid hyperplasia without features of neoplasm (Figure 1). This suggested amegakaryocytic thrombocytopenia and red cell aplasia, and the patient was started on oral cyclosporine at a daily dose of 6 mg/kg body weight. However, fourth day into therapy and he started to develop leukopenia with a WBC of 1,300/*μ*L and an absolute neutrophil count (ANC) of 700/*μ*L (normal 2,000–7,900/*μ*L) while his Hb (6.4 g/dL), Hct (18.4%), and platelet count (17,000/*μ*L) continued to be very low. The daily dose of oral cyclosporine was subsequently reduced to 3 mg/kg body weight, and he was started on subcutaneous filgrastim at a dose of 480 *μ*g daily. However, after nine days of cyclosporine therapy along with multiple platelet and red cell transfusions, the patient refused any further treatment or diagnostic procedures and left the hospital against medical advice. His last hematological parameters before discharge included a WBC of 3,900/*μ*L with an ANC of 1,400/*μ*L, Hb of 7.3 g/dL, Hct of 21.4%, and platelet count of 43,000/*μ*L.

## 3. Discussion

Thymoma, which accounts for nearly half of all tumors of the anterior mediastinum, originates within the epithelial cells of the thymus [5]. The thymus plays an important role in immune function, mainly with T-cell development. After migrating to the thymic cortex from the bone marrow, T-cell progenitors undergo sequences of positive and negative selections ensuring their recognition of foreign antigens and tolerance of self-peptides [6]. Neoplastic cells replace the normal epithelial cells in thymoma, and may disrupt T-cell maturation, and thus induct of self-tolerance [7]. This explains the great propensity of developing autoimmune disorders in thymoma, with the incidence reported being as high as 30% [6]. While myasthenia gravis is the most common autoimmune disorder associated; several autoimmune hematologic disorders like PRCA and aplastic anemia are also common [2–4, 8]. PRCA, thought to be due to T-cell-mediated destruction of erythroid progenitor cells, is reported in up to 5% of patients with thymoma [6, 9]. Thrombocytopenia, related to aplastic anemia or immune thrombocytopenic purpura, has been reported in patients with thymoma [10, 11]. Our patient with severe thrombocytopenia, however, had a bone marrow depleted of megakaryocytes and erythroid precursors with normal functioning myeloid precursors that differentiated it from aplastic anemia or immune thrombocytopenic purpura. These findings suggestive of concomitant PRCA and AAMT are rarely reported in thymoma [12–14].

AAMT is a rare disorder characterized by severe thrombocytopenia and scants megakaryocytes in the setting of otherwise normal bone marrow. Dysregulated humoral as well as cell-mediated immunity, consisting of antibodies against thrombopoietin and T-cell-mediated destruction on megakaryocytes, has been proposed as the possible pathogenesis [15, 16]. Thymomas can dysregulate the immune mechanism as described above and predispose a patient to AAMT. These AAMT in thymomas may be a very early presentation of impending aplastic anemia [14]. Though most AAMT cases do not respond to steroids or intravenous immunoglobulin, successful outcomes with various immunosuppressive therapies like cyclosporine, azathioprine, and rituximab are reported [17–21]. Successful treatment of both PRCA and AATM with cyclosporine in our patient would have consolidated similar findings in other reported cases of thymoma-associated PRCA and AATM [13, 22]. However, our case was complicated by rapidly developing leukopenia. This could represent the natural progression of the disease into aplastic anemia manifested as pancytopenia [14]. Furthermore, with our patient refusing further treatment after just nine days, the effectiveness, or lack thereof, of cyclosporine-based therapy could not be ascertained as prior reports had shown a treatment duration of 8 to 20 weeks before the hematological parameters stabilized [12, 13].

## 4. Conclusion

Acquired amegakaryocytic thrombocytopenia is a rare immune-mediated paraneoplastic syndrome seen in thymoma and can manifest years after the treatment of the primary tumor. AAMT may in fact be a very early presentation of impending aplastic anemia. Treating physicians need to be aware of this entity, more so given the need for specific immunosuppressive therapies in the background of apparent lack of response to steroids and intravenous immunoglobulin.
